# Fixational stability as a measure for the recovery of visual function in amblyopia

**DOI:** 10.1145/3450341.3458493

**Published:** 2021-05-25

**Authors:** Avi M. Aizenman, Dennis M. Levi

**Affiliations:** UC Berkeley, Vision Science, Berkeley, California, USA; UC Berkeley, Vision Science, Berkeley, California, USA

**Keywords:** eye tracking, amblyopia, development, eye movements

## Abstract

People with amblyopia have been shown to have decreased fixational stability, particularly those with strabismic amblyopia. Fixational stability and visual acuity have been shown to be tightly correlated across multiple studies, suggesting a relationship between acuity and oculomotor stability. Reduced visual acuity is the sine qua non of amblyopia, and recovery is measured by the improvement in visual acuity. Here we ask whether fixational stability can be used as an objective marker for the recovery of visual function in amblyopia. We tracked children’s fixational stability during patching treatment over time and found fixational stability changes alongside improvements in visual acuity. This suggests fixational stability can be used as an objective measure for monitoring treatment in amblyopia and other disorders.

## 1 INTRODUCTION

Amblyopia is a developmental visual disorder commonly associated with strabismus or anisometropia. Amblyopia is clinically important as it is the second most frequent cause of vision loss in infants (aside from refractive error) impacting 3–5% of the population [McKean-Cowdin et al. 2013; Sachsenweger 1968; Williams et al.2008]. Amblyopia leads to impaired visual acuity, contrast sensitivity, stereopsis, and form vision as well as oculomotor abnormalities including eccentric and unsteady fixation.

Recent work has shed a light on the importance of eye movements. Even at the level of small fixational saccades, these eye movements represent a critical stage of information processing, allowing the retina to begin extracting features [Rucci and Victor 2015]. The amblyopic eye shows reduced fixational stability, leading to increased drifts and subsequent corrective fixational saccades. However these corrective eye movements are error prone and tend to move the eye farther away from the intended fixation position [Chung et al. 2015]. This results in reduced fixational stability in the amblyopic eye, especially in strabismic amblyopia. Importantly, there is a significant correlation between the visual acuity and the fixational stability of the amblyopic eye [Chung et al. 2015; Shaikh et al. 2016; Subramanian et al. 2013]. It is currently unclear whether it is the unsteadiness of fixation that limits visual acuity, or visual acuity limiting fixational stability in amblyopic vision (a chicken and egg problem). Reduced visual acuity is the ‘sine qua non’ of amblyopia, and the degree to which visual acuity is reduced in the amblyopic eye directly relates to the depth and severity of a patients’ amblyopia. As such, it would be useful to establish whether changes in visual acuity with treatment in amblyopia are accompanied by changes to fixational stability. This relationship would establish whether fixational stability can be used as an objective measure for monitoring treatment in amblyopia and possibly other disorders. This is especially important because treatment of amblyopia is most effective in children, who may be too young to respond to subjective tests.

Ongoing research is exploring the use of specially designed dichoptic video games to facilitate treatment for amblyopia [Bavelier et al. 2010]. As these gamified treatments become more accessible, and eye tracking technology becomes less expensive and more prevalent, the possibility of using quantitative eye movement based metrics of a given treatment’s efficacy may prove to be very useful. Eye tracking may additionally allow for remote monitoring of patients in real time as they carry out their prescribed vision therapy. Additionally, using a non-verbal metric like fixational stability as a proxy for visual acuity is particularly useful for young children and toddlers with visual disorders that are unable to read an acuity chart.

## 2 METHODS

### 2.1 Experiment design

Each observer participated in 3 sessions over the course of 3 months. During each session clinical tests were performed and fixational stability was measured

#### 2.1.1 Participants.

Five children with amblyopia currently undergoing patching treatment or vision therapy (3 strabismic, 2 anisometropic, mean age 8.2 years) and five normally sighted children (mean age 9.4 years) participated in this study. Children with amblyopia were undergoing treatment, primarily patching the dominant eye, at the Meredith Morgan Eye Center at UC Berkeley. The experimenters were masked as to the treatment details.

#### 2.1.2 Clinical tests.

During each of the 3 sessions, visual acuity was measured using the Bailey Lovey acuity chart [Bailey and Lovie 1976]. Measures for stereopsis were taken using the interactive Asteroid test, which evaluates a wider range of stereoacuity than standard clinical tests [Vancleef et al. 2019].

#### 2.1.3 Measuring fixational stability.

To measure fixational stability, the dominant eye of each observer was patched and observers were asked to look at a 1° colorful smiley face on an otherwise black screen for 20-second intervals. There were ten 20-second trials in total and eye movements were recorded with an Eyelink II eye tracker tracking at 500 Hz.

## 3 RESULTS

### 3.1 Quantifying fixational stability

#### 3.1.1 Isoline Area.

To quantify fixational stability, we calculate the Isoline Area for each trial. We approximated the probability density function of eye positions using kernel density estimation [Węglarczyk 2018]. We then determined the area that corresponds to 68% of the highest density eye position traces. This measure makes no assumptions about the underlying distribution of eye position traces, unlike the commonly used Bivariate Contour Ellipse Area (BCEA), which assumes a Gaussian distribution of eye position [Castet and Crossland 2012].

#### 3.1.2 Identifying fixational saccades.

Fixational saccades were identified as samples with a velocity > 7° /sec and acceleration >350°/sec. We manually inspected all trials to remove any falsely-identified or missed fixational saccades.

### 3.2 Relationship between clinical measures and fixational stability

We computed correlations between fixational instability and clinical measures such as visual acuity (1A) and stereopsis (1B). We found that greater fixational instability was associated with worse visual acuity (*p*<0.001, Slope = 0.55±0.16 (95% CI: 0.23,0.88), r^2^ = 0.42) and deficits to stereopsis (*p*<0.01, Slope = 0.56 ± 0.14(95% CI: 0.27,0.85),r^2^ = 0.46).

### 3.3 Relationship between visual acuity improvements and fixational stability with treatment

**Figure 2:**
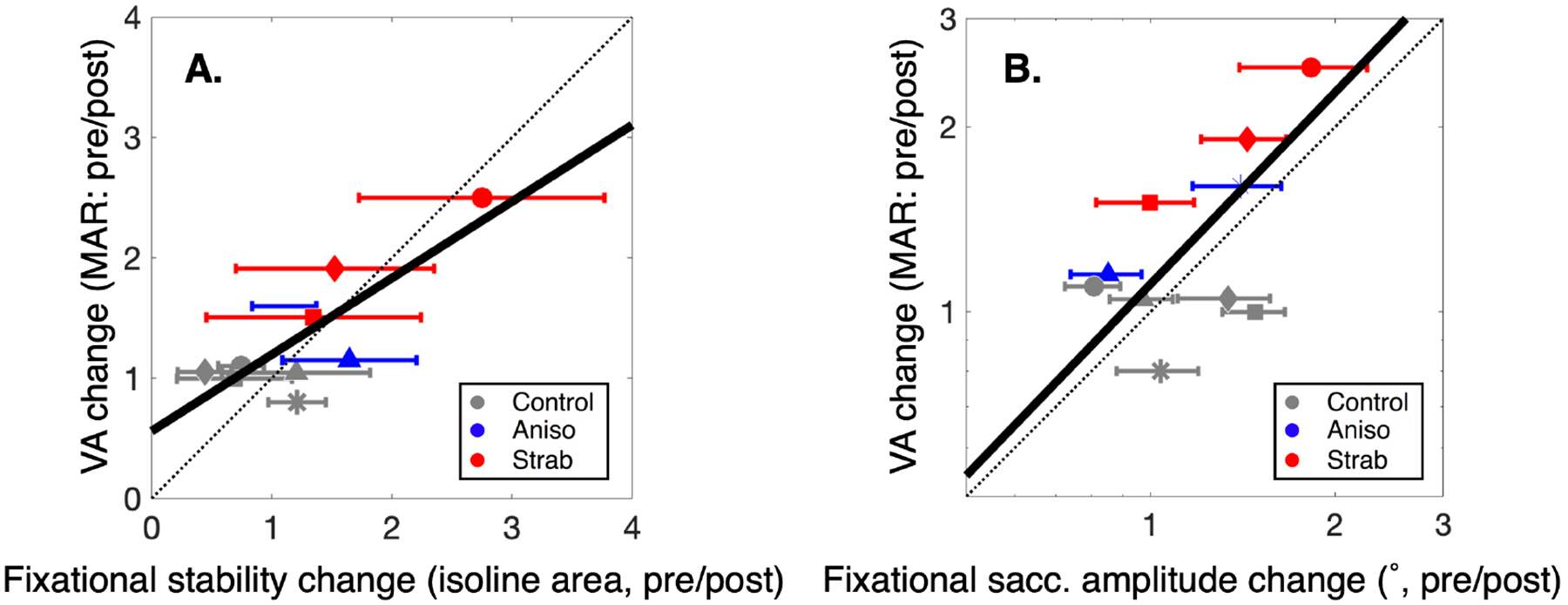
Correlation between changes in visual acuity as a function of fixational stability metrics for observers with strabismic (red) and anisometropic (blue) amblyopia, and normal vision controls (gray). In both plots change is represented as the pre/post ratio of measures taken (session 1/session 3). Error bars are RMS, and best fit lines are shown. A:Improvements in visual acuity with treatment are correlated with changes to fixational stability, and are associated with more stable fixation. B:Improvements in visual acuity with treatment are correlated with a decrease in fixational saccade amplitude.

In order to quantify changes between sessions, we calculated the pre/post ratio between session 1 and session 3 for both the clinical metrics and fixational stability measurements. A ratio of 1 means there was no change in that measure between session 1 and session 3. The change (pre/post) in isoline area showed a correlation with changes to visual acuity (*p*=0.03, Slope = 0.64 ± 0.18(95% CI: 0.23,1.04), r^2^ = 0.59), as shown in Fig 2A. As visual acuity in amblyopic eyes recovers with treatment, fixation becomes more stable, suggesting that the changes in fixational stability are tracking improvements to visual acuity. Current clinical treatment, aimed at improving visual acuity, consists primarily of patching the strong eye. However it is not clear whether improved fixation stability tracks improved visual acuity or vice-versa. We also found the changes to fixational stability and stereopsis are not correlated (Slope = 0.24, r^2^ = 0.03). In order to have fine stereopsis, good acuity is critical. However, even if both eyes have good acuity, stereopsis is not guaranteed. Patients with constant strabismus and good visual acuity in both eyes will typically be stereoblind [Levi et al. 2015]. Even with the recovery of visual function seen in observers with amblyopia, stereopsis is not recovering at a commensurate rate with fixational stability (by proxy visual acuity), which explains our findings.

These results suggest eye movement dynamics are changing with improvements to visual acuity. We considered whether changes to fixational saccade metrics such as amplitude, duration, error, and frequency are matched by improvements to visual acuity. Our analysis revealed a correlation between improvements to visual acuity and reduced fixational saccade amplitude (*p*<0.05, Slope = 1.07 ± 0.44(95% CI: 0.14,2.02), r^2^ = 0.43), as shown in the right panel of Fig 2B. As visual acuity improves with treatment, the amplitude of fixational saccades during attempted fixation decreases in the amblyopic eye. Importantly, the slope ≈ 1, implies that the improvement in visual acuity is proportional to the change in fixational saccade amplitude. No other comparisons between changes to fixational saccade metrics and changes in visual acuity showed a correlation.

### 3.4 Conclusion

In current best clinical practice, reduced visual acuity characterizes amblyopia. Considering that increased fixational unsteadiness in the amblyopic eye is correlated with poor visual acuity, it would be useful to establish whether improvements in visual acuity with treatment are accompanied by improved fixational stability in the amblyopic eye. Our preliminary results suggest that that visual acuity in the non-dominant eye and stereopsis are both correlated with fixational stability, replicating previous findings [Chung et al. 2015; Shaikh et al. 2016; Subramanian et al. 2013]. Importantly, we showed that with treatment, visual acuity in the amblyopic eye improves, and these improvements are correlated with increased fixational stability and smaller amplitudes during attempted fixaton. Interestingly, changes to fixational stability (and by proxy visual acuity) are not matched by changes to stereopsis, reflecting the inconsistent relationship between visual acuity and stereopsis [Levi et al. 2015]. Novel interventions for amblyopia that utilize virtual reality technology with eye tracking may consider monitoring fixational stability. This is a quick and easy way to collect an objective measure that tracks treatment efficacy, and these measurements can be collected remotely.

We are planning to collect more data for this experiment in the future. Previous work has suggested fixation is particularly unstable in the case of strabismic amblyopia [Chung et al. 2015; Subramanian et al. 2013]. We plan to look at differences in recovery and fixational stability dynamics between observers with strabismic or anisometropic amblyopia. It is also unclear if fixational stability is the limiting factor on acuity or vice versa. We also plan to assess the temporal dynamics of treatment and whether improvements to fixational stability are seen first, and visual acuity follows or vice versa. This will help inform the chicken and egg problem surrounding fixational stability and acuity.

## Figures and Tables

**Figure 1:**
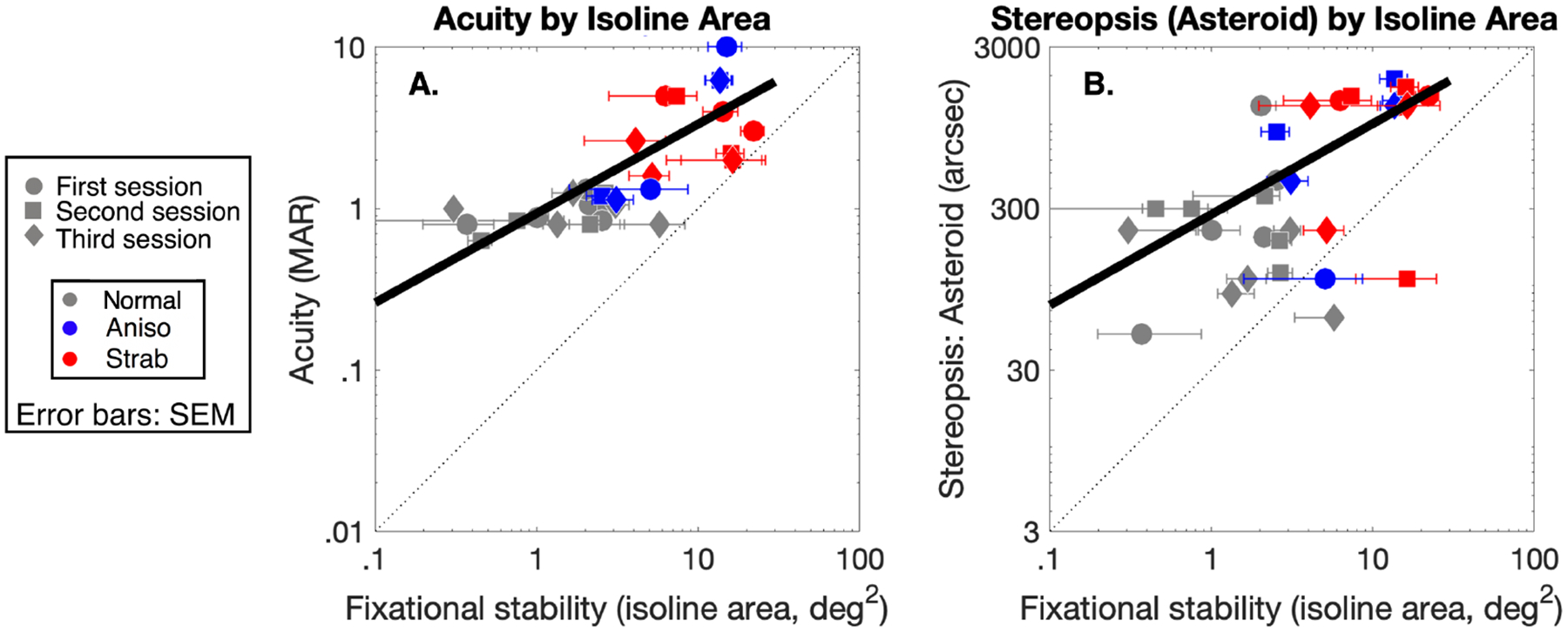
Correlation between clinical measures and fixational stability for observers with strabismic (red) and anisometropic (blue) amblyopia, and normal vision controls (gray). Each data point represents measurements from a single session. Error bars are SEM, and best fit lines are shown. A:Visual acuity in MAR by fixational stability (Isoline Area). Larger values correspond to worse visual acuity and less stable fixation. B: Stereopsis in arcsec by fixational stability (Isoline Area). Larger values correspond to worse stereopsis and less stable fixation. Visual acuity and stereopsis show a correlation with fixational stability.

## CCS CONCEPTS

• Computing methodologies → Perception; Virtual reality.

## References

[R1] Bailey Ian L and Lovie Jan E. 1976. New design principles for visual acuity letter charts. American journal of optometry and physiological optics 53, 11 (1976), 740–745. 998716 10.1097/00006324-197611000-00006

[R2] Bavelier Daphne, Levi Dennis M, Li Roger W, Dan Yang, and Hensch Takao K. 2010. Re-moving brakes on adult brain plasticity: from molecular to behavioral interventions. Journal of Neuroscience 30, 45 (2010), 14964–14971. 21068299 10.1523/JNEUROSCI.4812-10.2010 PMC2992973

[R3] Castet Eric and Crossland Michael. 2012. Quantifying eye stability during a fixation task: a review of definitions and methods. Seeing and Perceiving 25, 5 (2012), 449–469. 22370759 10.1163/187847611X620955

[R4] Chung Susana TL, Kumar Girish, Li Roger W, and Levi Dennis M. 2015. Characteristics of fixational eye movements in amblyopia: Limitations on fixation stability and acuity? Vision research 114 (2015), 87–99. 25668775 10.1016/j.visres.2015.01.016 PMC4529398

[R5] Levi Dennis M, Knill David C, and Bavelier Daphne. 2015. Stereopsis and amblyopia:A mini-review. Vision research 114 (2015), 17–30. 25637854 10.1016/j.visres.2015.01.002 PMC4519435

[R6] McKean-Cowdin Roberta, Cotter Susan A, Tarczy-Hornoch Kristina, Wen Ge, Kim Jeniffer, Borchert Mark, Varma Rohit, Multi-Ethnic Pediatric Eye Disease Study Group, 2013. Prevalence of amblyopia or strabismus in asian and non-Hispanic white preschool children: multi-ethnic pediatric eye disease study. Ophthalmology 120, 10 (2013), 2117–2124. 23697956 10.1016/j.ophtha.2013.03.001 PMC4848013

[R7] Rucci Michele and Victor Jonathan D. 2015. The unsteady eye: an information-processing stage, not a bug. Trends in neurosciences 38, 4 (2015), 195–206. 25698649 10.1016/j.tins.2015.01.005 PMC4385455

[R8] Sachsenweger R. 1968. Problems of organic lesions in functional amblyopia. In International strabismus symposium. Karger Publishers, 63–66.

[R9] Shaikh Aasef G, Otero-Millan Jorge, Kumar Priyanka, and Ghasia Fatema F. 2016. Abnormal fixational eye movements in amblyopia. PLoS One 11, 3 (2016), e0149953. 26930079 10.1371/journal.pone.0149953 PMC4773232

[R10] Subramanian Vidhya, Jost Reed M, and Birch Eileen E. 2013. A quantitative study of fixation stability in amblyopia. Investigative ophthalmology & visual science 54, 3 (2013), 1998–2003. 23372053 10.1167/iovs.12-11054 PMC3604910

[R11] Vancleef Kathleen, Serrano-Pedraza Ignacio, Sharp Craig, Slack Gareth, Black Carla, Casanova Therese, Hugill Jess, Rafiq Sheima, Burridge James, Puyat Vito, 2019. ASTEROID: a new clinical stereotest on an autostereo 3D tablet. Translational vision science & technology 8, 1 (2019), 25–25. 10.1167/tvst.8.1.25 PMC6396686 30834173

[R12] Węglarczyk Stanisław. 2018. Kernel density estimation and its application. In ITM Web of Conferences, Vol. 23. EDP Sciences.

[R13] Williams Cathy, Northstone Kate, Howard Margaret, Harvey Ian, Harrad RA, and Sparrow JM. 2008. Prevalence and risk factors for common vision problems in children: data from the ALSPAC study. British Journal of Ophthalmology 92, 7 (2008), 959–964. 18480306 10.1136/bjo.2007.134700

